# Overexpression of Cytoplasmic *Tc*SIR2RP1 and Mitochondrial *Tc*SIR2RP3 Impacts on *Trypanosoma cruzi* Growth and Cell Invasion

**DOI:** 10.1371/journal.pntd.0003725

**Published:** 2015-04-15

**Authors:** Carla Ritagliati, Victoria L. Alonso, Romina Manarin, Pamela Cribb, Esteban C. Serra

**Affiliations:** 1 Instituto de Biología Molecular y Celular de Rosario (IBR), Consejo Nacional de Investigaciones Científicas y Técnicas, Rosario, Argentina; 2 Facultad de Ciencias Bioquímicas y Farmacéuticas, Universidad Nacional de Rosario (UNR), Rosario, Argentina; Liverpool School of Tropical Medicine, UNITED KINGDOM

## Abstract

**Background:**

*Trypanosoma cruzi* is a protozoan pathogen responsible for Chagas disease. Current therapies are inadequate because of their severe host toxicity and numerous side effects. The identification of new biotargets is essential for the development of more efficient therapeutic alternatives. Inhibition of sirtuins from *Trypanosoma brucei* and *Leishmania* ssp. showed promising results, indicating that these enzymes may be considered as targets for drug discovery in parasite infection. Here, we report the first characterization of the two sirtuins present in *T*. *cruzi*.

**Methodology:**

Dm28*c* epimastigotes that inducibly overexpress *Tc*SIR2RP1 and *Tc*SIR2RP3 were constructed and used to determine their localizations and functions. These transfected lines were tested regarding their acetylation levels, proliferation and metacyclogenesis rate, viability when treated with sirtuin inhibitors and *in vitro* infectivity.

**Conclusion:**

*Tc*SIR2RP1 and *Tc*SIR2RP3 are cytosolic and mitochondrial proteins respectively. Our data suggest that sirtuin activity is important for the proliferation of *T*. *cruzi* replicative forms, for the host cell-parasite interplay, and for differentiation among life-cycle stages; but each one performs different roles in most of these processes. Our results increase the knowledge on the localization and function of these enzymes, and the overexpressing *T*. *cruzi* strains we obtained can be useful tools for experimental screening of trypanosomatid sirtuin inhibitors.

## Author Summary

Sirtuins are a family of deacetylases, evolutionary conserved from bacteria to mammals. They participate in the regulation of a wide range of nuclear, cytoplasmic and mitochondrial pathways, and are considered pro-life enzymes. In the last years the search for sirtuin inhibitors was a very active field of research, with potential applications in a large number of pathologies, including parasitic diseases. We are interested in the study of the two sirtuins present in the protozoan parasite *Trypanosoma cruzi*, being our objective to understand their function. First, we determined the localization of these enzymes in the parasite: *Tc*SIR2RP1 is a cytoplasmic enzyme and *Tc*SIR2RP3 localizes in the mitochondrion. When we overexpress cytoplasmic *Tc*SIR2RP1, the transgenic parasites differentiate to metacyclic trypomastigotes and infect mammalian cells more efficiently. In contrast, the overexpression of mitochondrial *Tc*SIR2RP3 does not affect metacyclogenesis but modifies epimastigotes growth and slightly increases the proliferation of the parasite in the intracellular stage. We also used these transgenic lines to test their sensibility to previously described sirtuin inhibitors.

## Introduction

Acetylation is a ubiquitous protein modification present in prokaryotic and eukaryotic cells that participates in the regulation of many cellular processes. A limited set of acetyltransferases and deacetylases, and of the acetyl-lysine “reading” domain (bromodomain) are the principal components of the acetylation/deacetylation machinery. Among them, protein deacetylases are enzymes that catalyze the removal of acetyl groups from the ε-amino group of lysine residues and are classified into four classes. Sirtuins, the class III (NAD^+^-dependent) protein deacetylases, are homologous to the yeast transcriptional repressor, Sir2 [1]. Sir2, as well as all sirtuins, deacetylates lysine residues in a unique chemical reaction that consumes nicotinamide adenine dinucleotide (NAD^+^) and generates nicotinamide, O-acetyl-ADP-ribose (OAADRr), and the deacetylated substrate [2].

*Saccharomyces cerevisiae* Sir2, the founding member of the group, is a histone deacetylase (reviewed in [3]) involved in a range of chromatin-mediated processes; namely, gene silencing at telomeres and mating-type loci, DNA repair [4–5], suppression of recombination within ribosomal DNA (rDNA)[6], DNA replication [7], chromosome stability [8] and plasmid segregation [9]. However, the identification and characterization of new members of this protein family in other organisms led to the discovery of more diverse functions and localizations. It is now recognized that sirtuins remove acetyl groups from lysines in nuclear, cytosolic and mitochondrial protein substrates [10]. Sirtuins are evolutionarily conserved enzymes present in all kingdoms of life, ranging from bacteria to higher eukaryotes including humans. Members of this family share a core domain of ~250 amino acids that exhibits 25–60% sequence identity between different organisms. Genes coding for seven sirtuins (SIRT 1–7) have been found in the human genome, with subcellular distribution, substrate specificity, and cellular functions quite diverse [11].

*Trypanosoma cruzi* is a hemoflagellate protozoan parasite, branched early from the eukaryal lineage. It is an intracellular pathogen responsible for Chagas’ disease, or American Trypanosomiasis, a chronic infectious disease affecting 8 million people [12]. While Chagas disease is endemic in Latin America, a significant increase in confirmed cases of Chagas has recently been reported in the USA, Canada, Japan, Australia and Europe, indicating that it is an emerging disease [13]. Current therapies rely on a very small number of drugs, most of which are inadequate because of their severe host toxicity and numerous side effects. The identification of new biotargets is essential for the development of more efficient therapeutic alternatives. The structural basis for inhibition of sirtuins has been established through previous structural and functional studies [14–17]. Involvement of sirtuins in the cell cycle strongly suggests a role for these enzymes in cancer and the potential use of their inhibitors as anticancer drugs [18]. In addition, inhibition of sirtuins from *Trypanosoma brucei* and *Leishmania* ssp. showed promising results, indicating that these enzymes may be considered as targets for drug discovery in parasite infection [19–22].

*T*. *cruzi* belongs to the Kinetoplastida order, Trypanosomatidae family, as well as *Trypanosoma brucei* and *Leishmania* ssp., and together they are termed TriTryps. Genes encoding three Sir2 related proteins (SIR2RPs) were found in the TriTryps. The trypanosomatid genes were designated SIR2-related proteins, SIR2RP1–3. A previous phylogenetic analysis places SIR2RP1 in a group with *Sc*Sir2, *Hs*SIRT1 and *Hs*SIRT2, while SIR2RP2 and SIR2RP3 are more closely related to bacterial proteins and to *Hs*SIRT4 and *Hs*SIRT5 respectively [23]. However, a more recent extensive analysis places SIR2RP1 in the sirtuins subgroup Ib, together with cytoplasmic *Hs*SIRT2, and is now clearly differentiated from the nuclear *Hs*SIRT1 [24].

SIR2RP1 from several *Leishmania* species and all three SIR2RPs from *T*. *brucei* have been characterized [16, 23]. SIR2RP1 is found in cytoplasmic granules in different stages of *L*. *major*, *L*. *infantum* and *L*. *amazonensis*; and, under certain conditions, it is observed in the excreted/secreted fraction [25–27]. *Li*SIR2RP1 is also found associated with the cytoskeleton network and deacetylates α-tubulin, a function that resembles that of human SIRT2 and HDAC6. In contrast, *Tb*SIR2RP1 is a nuclear chromosome-associated protein. It is expressed throughout *T*. *brucei* life cycle, catalyses NAD^+^-dependent ADP ribosylation and deacetylation of histones and in the mammalian-infective bloodstream-stage controls DNA repair and repression of RNA polymerase I-mediated expression immediately adjacent to telomeres [16, 23]. *Tb*SIR2RP2 and 3 localize in the single mitochondrion of the parasite and it was reported that their interference do not produce growth or differentiation defects. In *Trypanosoma cruzi*, the gene coding for SIR2RP2 is lacking. This general landscape suggests that in spite of their sequence similarity, sirtuin variants from TriTryps have evolved to different functions.

Here, we report the first characterization of the two sirtuins present in *T*. *cruzi*. In epimastigotes, *Tc*SIR2RP1 localizes in the cytoplasm while *Tc*SIR2RP3 localizes to the parasite’s single mitochondrion. Overexpression of *Tc*SIR2RP1 causes no alteration to epimastigote growth, but it increases the number of trypomastigotes obtained in *in vitro* metacyclogenesis and the infectivity rate of Vero cells. In contrast, overexpression of *Tc*SIR2RP3 slightly decreases epimastigote growth and the infectivity rate of Vero cells, it does not affect the *in vitro* differentiation to metacyclic trypomastigotes, and it increases the proliferation rate of intracellular amastigotes. Finally, overexpression of either of these sirtuins protects the parasite from the effect of sirtuin inhibitors.

## Materials and Methods

### Ethics statement

All experiments were approved by the Institutional Animal Care and Use Committee of the School of Biochemical and Pharmaceutical Sciences, National University of Rosario (Argentina) (File 6060/227) and conducted according to specifications of the US National Institutes of Health guidelines for the care and use of laboratory animals. Rabbits were only used for the production of polyclonal antibodies. The rabbits were immunized three times with the protein and an equal volume of Freund´s adjuvant, and bled two weeks after the final injection [28].

### Molecular cloning of *T*. *cruzi* sirtuins

*Tc*SIR2RP1 and *Tc*SIR2RP3 genes were amplified using the following oligonucleotides: SIR2RP1ss (5’ AAAGGATCCATGAATCAAGATAACGCCAAC), SIR2RP1**HA**as (5’ AACTCGAG **AGCATAATCCGGCACATCATACGGATA**TTTTCGGT CTGTCAG), SIR2RP3ss (5’ AAGGATCCATGAAGCCGCGGCGTCAGAT) and SIR2RP3HAas (5′ AACTCGAG **AGCATAATCCGGCACATCATACGGATA**CACCGCGT CTTGAAG). DNA purified from *T*. *cruzi* epimastigotes was used as template. The PCR products obtained with a proofreading DNA polymerase were inserted into pCR2.1-TOPO vector (Invitrogen) and sequenced. *Tc*SIR2RP1 and *Tc*SIR2RP3 coding regions were inserted into a pENTR3C vector (Gateway system Invitrogen) using the *Bam*HI/*Xho*I restriction sites included in the oligonucleotides (underlined) and then transferred to pDEST17 (Gateway system Invitrogen) and p*Tc*INDEX-GW vectors by recombination using LR clonase II enzyme mix (Invitrogen). The pDEST17 constructs were transformed into *Escherichia coli* BL21 pLysS and recombinant proteins, fused to a six histidine tag, were obtained by expression-induction with 0.5 mM IPTG for 5 h at 37°C. The proteins were purified by affinity chromatography using a Ni-NTA agarose resin (Qiagen) following the manufacturer’s instructions.

### Polyclonal antibodies

Rabbit polyclonal antisera against *Tc*SIR2RP1 and *Tc*SIR2RP3 were obtained by inoculating subcutaneously the recombinant proteins to these animals as described above.

### Parasite cultures

*T*. *cruzi* epimastigote forms (Dm28*c* strain) were cultured at 28°C in liver infusion tryptose (LIT) medium (5 g/L liver infusion, 5 g/L bacto-tryptose, 68 mM NaCl, 5.3 mM KCl, 22 mM Na_2_HPO_4_, 0.2% (w/v) glucose and 0.002% (w/v) hemin) supplemented with 10% (v/v) heat-inactivated FCS, 100 U/ml penicillin and 100 mg/l streptomycin. Cell viability was assessed by direct microscopic examination.

### Transfection of epimastigotes

For inducible expression of Sir2rp1-3 genes in the parasite, we first generated a cell line expressing T7 RNA polymerase and tetracycline repressor genes by transfecting epimastigotes with the plasmid pLew13 using a standard electroporation method. Briefly, epimastigote forms of *T*. *cruzi* Dm28*c* were grown at 28°C in LIT medium, supplemented with 10% FCS, to a density of approximately 3 × 10^7^ cells/ml. Parasites were then harvested by centrifugation at 2,000 × g for 5 min at room temperature, washed once in PBS and resuspended in 0.35 ml of transfection buffer pH 7.5 (0.5 mM MgCl_2_, 0.1 mM CaCl_2_ in PBS) to a density of 1 × 10^8^ cells/ml. Cells were then transferred to a 0.2 cm gap cuvette (Bio-Rad) and ~50 μg of DNA was added in a final volume of 40 μl. The mixture was placed on ice for 15 min and then subjected to 2 pulses of 450 V and 500 μF using GenePulser II (Bio-Rad, Hercules, USA). After electroporation, cells were transferred into 3 ml of LIT medium containing 10% FCS, maintained at room temperature for 15 minutes and then incubated at 28°C. Geneticin (G418; Life Technologies) was added at a concentration of 200 μg/ml, and parasites were incubated at 28°C. After selection, transfected epimastigotes were grown in the presence of 200 μg/ml of G418. This parental cell line was then transfected with p*Tc*INDEX-GW constructs and transgenic parasites were obtained after 3 weeks of selection with 100 μg/ml G418 and 200 μg/ml Hygromycin B (Sigma).

### *In vitro* metacyclogenesis

To obtain metacyclic trypomastigotes, epimastigotes were differentiated *in vitro* following the procedure described by Contreras and coworkers [29] using chemically defined conditions (TAU3AAG medium). Briefly, cells were washed with PBS and incubated in TAU medium (190 mM NaCl, 17 mM KCl, 2 mM MgCl_2_, 2mM CaCl_2_, 8 mM phosphate buffer pH 6.0) in the absence or presence of 0.25 μg/ml Tetracycline, reaching a density of 5 x 10^8^ parasites/ml at 28°C for 2 hours. Then they were diluted 1:100 in TAU3AAG Medium (TAU medium plus 10 mM Glucose, 2 mM L-Aspartic Acid, 50 mM L-Glutamic Acid and 10 mM L-Proline) and incubated at 28°C for 72 hours, again in the absence or presence of Tetracycline. Finally, the parasites were fixed, stained with Giemsa, visualized with a Nikon Eclipse Ni-U microscope and counted using ImageJ software [30]. Only parasites with a fully elongated nucleus and a round kinetoplast at the posterior portion end of the parasite were considered metacyclic forms [31]. Five hundred parasites from each triplicate were counted and the experiment was repeated tree times.

### *Trypanosoma cruzi* infection of Vero cells

Vero cells were cultured in DMEM medium (Life Technologies), supplemented with 2 mM L-glutamine, 10% FCS, 100 U/ml penicillin and 100 μg/ml streptomycin.

Metacyclic trypomastigotes were obtained by spontaneous differentiation of epimastigotes at 28°C. Cell-derived trypomastigotes were obtained by infection with metacyclic trypomastigotes of Vero cell monolayers. After two rounds of infections, the cell-derived trypomastigotes were used for the infection and intracellular amastigotes proliferation experiments. Trypomastigotes were collected by centrifugation of the supernatant of previously infected cultures at 2,000 x g at room temperature for 10 minutes and incubated for 3 hours at 37°C in order to allow the trypomastigotes to move from the pellet into the supernatant. After this period, the supernatant was collected and trypomastigotes were counted in a Neubauer chamber. The purified trypomastigotes were pre-incubated in the presence or absence of 0.25 μg/ml Tetracycline for 3 hours and then used to infect new monolayers of Vero cells at a ratio of 10 parasites per cell. After 6 h of infection at 37°C, the free trypomastigotes were removed by successive washes using saline solution. Cultures were incubated in complete medium with or without Tetracycline (0.25 μg/ml) for 2 days post-infection. Infections were performed in DMEM supplemented with 2% FCS. Cells were then fixed in methanol and the percentage of infected cells and the mean number of amastigotes per infected cell, were determined by counting the slides after Giemsa staining using a Nikon Eclipse Ni-U microscope, by counting ~1000 cells per slide. The significances of the results were analyzed by a two-way ANOVA using GraphPad Prism version 6.0 for Mac. Results are expressed as means ± SEM of triplicates, and represent one of three independent experiments performed.

### Protein extracts

Exponentially growing epimastigotes were washed twice with cold PBS, pellets were resuspended in urea lysis buffer (8 M Urea, 20 mM Hepes pH 8, 1 mM phenylmethylsulphonyl fluoride (PMSF), and Protease Inhibitor Cocktail set I, Calbiochem), incubated at room temperature for 20 minutes and boiled for 5 minutes with protein loading buffer. Insoluble debris was eliminated by centrifugation. The same procedure was applied to amastigote and trypomastigote cellular pellets.

### Subcellular fractionation by differential centrifugation

Transfected Dm28*c* epimastigotes in exponential growth phase were centrifuged for 10 min at 2,000 x g and washed twice in homogenization buffer (25 mM Tris-HCl pH 8, 1 mM EDTA, 0.25 M sucrose, 1 mM PMSF). Subcellular fractions were obtained following the procedure described by Opperdoes and coworkers [32]. The parasites were grinded in a pre-chilled mortar with 1 x wet weight silicon carbide until no intact cells were observed under the light microscope. The lysate was diluted and centrifuged at 100 x g for 10 min to remove the silicon carbide. Unbroken cells, nuclei and debris were sedimented at 1,000 x g for 10 min (Fraction N). From the resulting soluble extract a large-granule fraction (LG) was separated at 5,000 x g for 15 min, a small-granule fraction (SG) at 20,000 x g for 20 min and microsomal fraction (M) at 139,000 x g for 1 h. All the sediments were resuspended in urea lysis buffer.

### Western blot

Protein extracts were fractioned in SDS-PAGE and transferred to nitrocellulose membranes. Transferred proteins were visualized with Ponceau S. Membranes were treated with 10% non-fat milk in PBS for 2 hours and then incubated with specific antibodies diluted in PBS for 3 hours. Antibodies used were: rat monoclonal anti-HA (ROCHE), rabbit polyclonal anti-*Tc*SIR2RP1 and anti-*Tc*SIR2RP3, rabbit polyclonal anti-Acetyl-lysine (Millipore), mouse monoclonal anti-acetylated α-tubulin clone 6-11B-1 (Sigma Aldrich), mouse monoclonal anti-trypanosome α-tubulin clone TAT-1, rabbit polyclonal anti-*T*. *cruzi* mitochondrial Malate Dehydrogenase (*Tc*MDHm), rabbit and mouse polyclonal anti-*T*. *cruzi* Tyrosine Amine Transferase (*Tc*TAT), mouse polyclonal anti-*T*. *cruzi* Aspartate Transaminase (*Tc*ASAT) and rabbit polyclonal anti-*T*. *cruzi* Bromodomain Factor 2 (*Tc*BDF2). Bound antibodies were detected using peroxidase labeled anti-mouse, anti-rabbit IgGs (GE Healthcare) or anti-rat IgG (Thermo Scientific) and developed using ECL Prime kit (GE Healthcare) according to manufactures protocol.

### Immunocytolocalization

Trypomastigotes and exponentially growing epimastigotes were centrifuged, washed twice in PBS, settled on polylisine-coated coverslips and fixed with 4% para-formaldehyde in PBS at room temperature for 20 minutes. For the mitochondrial staining, parasites were resuspended in PBS and incubated with 1 μM MitoTracker (Invitrogen) for 30 minutes at 28°C, washed twice in PBS and fixed with 4% para-formaldehyde. Fixed parasites were washed with PBS and permeabilized with 0.1% Triton X-100 in PBS for 10 minutes. After washing with PBS, parasites were incubated with the appropriate primary antibody diluted in 5% BSA in PBS for 2 hours at room temperature. In colocalization experiments both antibodies were incubated together. Non-bound antibodies were washed with 0.01% Tween 20 in PBS and then the slides were incubated with fluorescent-conjugated anti-mouse (FITC, Jackson Immuno Research) or anti-rat (FITC, Life Technologies) and anti-rabbit (Cy3, Life Technologies) IgG antibodies and 2 μg/ml of DAPI for 1 hour. The slides were washed with 0.01% Tween 20 in PBS and finally mounted with VectaShield (Vector Laboratories). To analyze intracellular amastigotes, Vero cells monolayers were grown on coverslips and infected with *T*. *cruzi* trypomastigotes as described above. Two days post-infection cultures were washed with PBS and fixed with methanol at room temperature for 3 minutes. The same procedure described above was followed for immunodetection. Images were acquired with a confocal Nikon Eclipse TE-2000-E2 microscope using Nikon EZ-C1 Software. Adobe Photoshop CS and ImageJ software were used to process all images.

### Treatment of *T*. *cruzi* epimastigotes with sirtuin inhibitors

To determine the IC_50_ values of the sirtuin inhibitors, epimastigotes of *T*. *cruzi* Dm28*c* strain were cultured at 28°C in liver infusion tryptose medium (LIT) supplemented with 10% FCS in the absence or presence of Nicotinamide, Cambinol and Ex-527 (Sigma) at various concentrations, in triplicates. Cell growth was determined after culture for 72 hours by counting viable forms in an automatized hemocytometer adapted to count epimastigotes (WL 19 Counter AA, Weiner Lab). Then, Dm28*c* wt, Dm28*c* p*Tc*INDEXGW-SIR2RP1HA and Dm28*c* p*Tc*INDEXGW-SIR2RP3HA strains (uninduced and induced with 0.5 μg/ml Tetracycline), were cultured at 28°C in LIT with FCS in the absence or presence of the sirtuin inhibitors at concentrations above their IC_50_ values.

### Statistical analysis

Experiments were performed in triplicate, and at least three independent experiments were performed. Data are presented as the mean ± SEM. Statistical analysis of the data was carried out using two-way ANOVA and unpaired Mann-Whitney, two-tailed Student t test. Differences between the experimental groups were considered significant as follows: p<0.05 (*), p<0.005 (**), p<0,001 (***) and p<0.0001 (****). To determine the IC_50_ values, we used nonlinear regression on Prism 6.0 GraphPad software. Student’s t test was applied to ascertain the statistical significance of the observed differences in the IC_50_ values.

## Results

### Identification of Sir2 homologs in *Trypanosoma cruzi*

Two protein coding sequences (TcCLB.507519.60 and TcCLB.506559.80) corresponding to Sir2 related proteins were identified in the *T*. *cruzi* genome, termed *Tc*SIR2RP1 and *Tc*SIR2RP3 respectively (http://www.tritrypdb.org/tritrypdb/). *Tc*Sir2rp1 and *Tc*Sir2rp3 encode proteins of 359 and 241 amino acids, with predicted molecular weights of ~ 39.6 and 26.8 kDa and pIs of 6.39 and 6.51, respectively. The alignment of *T*. *cruzi* sirtuins with human SIRTs and *Sc*Sir2 (S1 Fig) shows that although *Tc*SIR2RPs lack the N-terminal portion, which is required for nucleolar localization in *Sc*Sir2, they contain a complete catalytic domain (Pfam: PF02146). SIR2RP1 contains a Serine-rich motif towards the C-terminus and one of the Cys residues from the zinc-binding motif (CX_2_CX_20_CX_2_C type) is absent in SIR2RP3. The GAD and NID motifs as well as other residues important for catalysis are conserved (HG, arrowheads in S1 Fig). The catalytic domain of *Tc*SIR2RP1 and *Tc*SIR2RP3 share a sequence identity/similarity of 17.5%/26.3% with each other and 23.1%/33.9% and 20.8%/ 33.7% with *Sc*SIR2 respectively. Similarly to what Greiss and Gartner observed [24], *Tc*SIR2RP1 grouped with the cytoplasmatic human SIRT2 whereas *Tc*SIR2RP3 is more related to mitochondrial *Hs*SIRT5 and bacterial sirtuins (S2 Fig). Despite the discrepancies observed for each sirtuin from different Tritryp species regarding their localization and function, they seem to be conserved at the sequence level.

**Fig 1:**
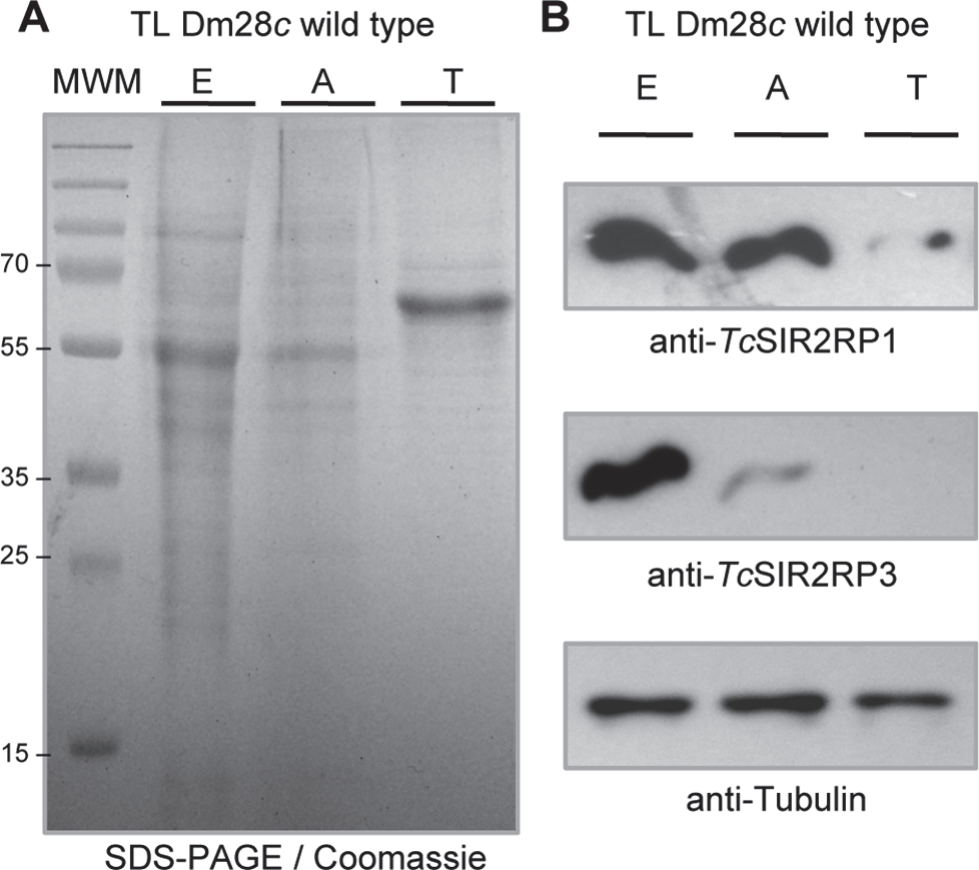
The expression of sirtuins is developmentally regulated. Equal amounts of parasite lysate from epimastigotes (E), amastigotes (A) and trypomastigotes (T) were loaded on SDS-PAGE followed by (A) Coomassie staining or (B) western blot analysis using the following antibodies: anti-*Tc*SIR2RP1, anti-*Tc*SIR2RP3 and anti-Tubulin as load control.

In order to evaluate *Tc*SIR2RP1 and *Tc*SIR2RP3 expression in *T*. *cruzi*, antibodies were raised against the recombinant proteins and purified by affinity chromatography. After confirming the specificity of the antibodies (S3 Fig showed a single band of the expected molecular weights), they were used in Western blots to test total lysates of epimastigotes, amastigotes and trypomastigotes. As can be observed in Fig 1, the expression of sirtuins is developmentally regulated throughout *T*. *cruzi* life cycle. *Tc*SIR2RP1 shows similar expression levels in epimastigotes and amastigotes, but lower in trypomastigotes. *Tc*SIR2RP3 expression levels are higher in epimastigotes than in amastigotes and it is not detected in trypomastigotes under the conditions assayed.

### Inducible expression of sirtuins in *T*. *cruzi*

**Fig 2:**
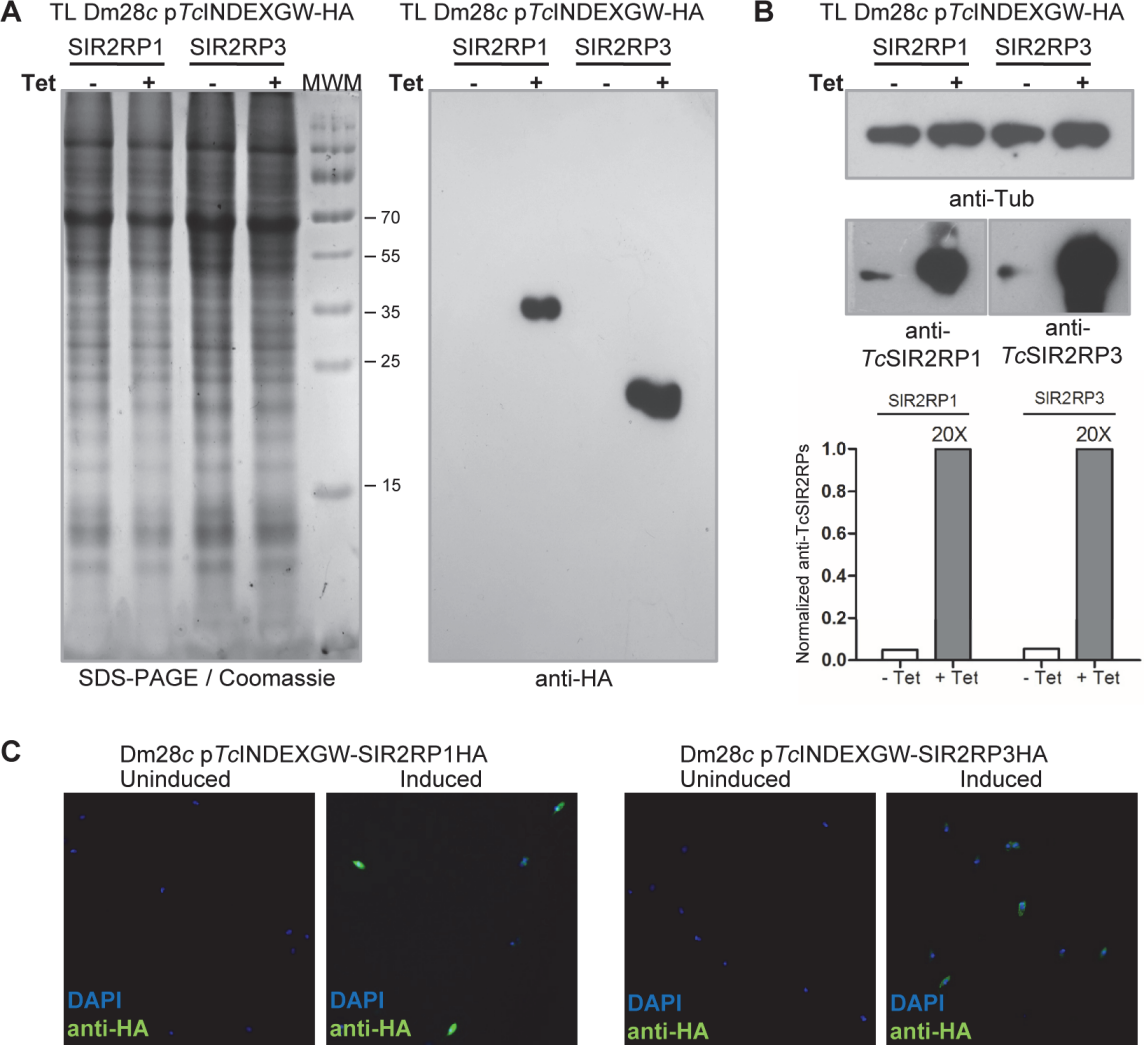
Inducible expression of sirtuins in epimastigotes. Equal amounts of parasite total lysate from each line (p*Tc*INDEXGW-SIR2RP1HA and p*Tc*INDEXGW-SIR2RP3HA) in the absence (-) or presence (+) of 0.25 μg/ml Tetracycline for 24 hours, were loaded on SDS-PAGE and stained with Coomassie (left panel), followed by western blot analysis using rat anti-HA monoclonal antibodies (A), or mouse anti-Tubulin and specific rabbit polyclonal antibodies against *Tc*SIR2RP1 and *Tc*SIR2RP3 (B). The degree of overexpression observed with the specific antibodies were quantified and normalized to α-tubulin intensity. (C) Immunofluorescence microscopy of uninduced and induced (0.25 μg/ml Tetracycline, 24 hours) parasites using rat anti-HA and FITC-conjugated anti-rat antibodies (green). DNA was stained with DAPI (blue).

**Fig 3:**
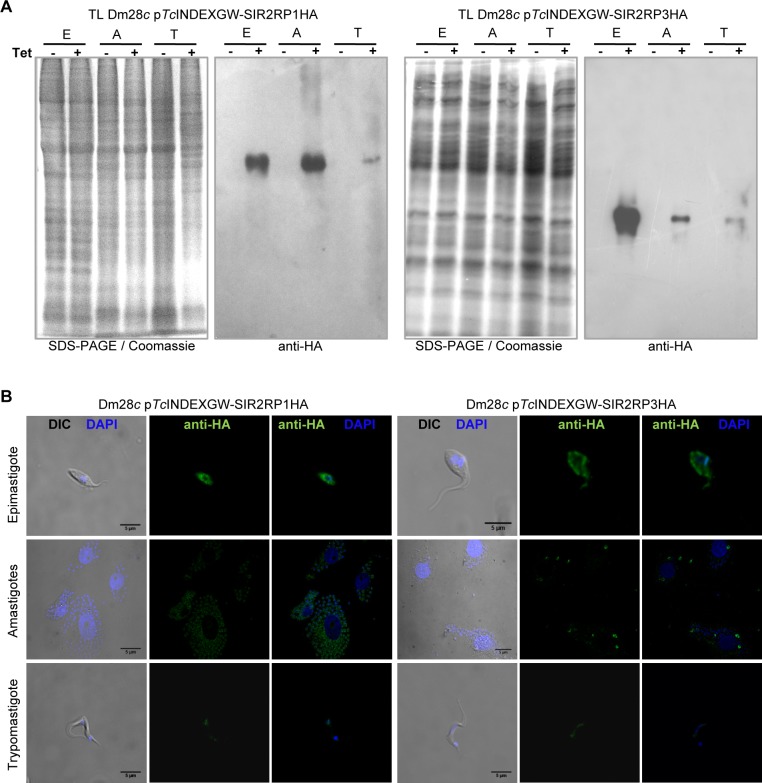
Sirtuins inducible expression throughout *T*. *cruzi* life cycle. (A) Western blot analysis. Equal amounts of parasite lysate from epimastigotes (E), amastigotes (A) and trypomastigotes (T) in the absence (-) or presence (+) of 0.25 μg/ml Tetracycline for 24 hours were loaded on SDS-PAGE followed by western blot analysis using rat anti-HA monoclonal antibodies. (B) Immunofluorescence confocal microscopy of induced (0.25 μg/ml Tetracycline, 24 hours) parasites using rat anti-HA, FITC-conjugated anti-rat antibodies (green), and DAPI (blue). Scale bar: 5 μm.

Overexpression of *Tc*SIR2RP1 and *Tc*SIR2RP3 enzymes was performed using the *T*. *cruzi* inducible vector p*Tc*INDEXGW [33]. Epimastigote cell lines expressing each sirtuin with a C-terminal HA tag under the control of a Tetracycline-regulated promoter were generated (Materials and Methods). The induction of the expression by Tetracycline was tested by western blot (Fig 2A and 2B) and immunofluorescense (Fig 2C). Western blot analysis of whole-cell extracts with rat monoclonal anti-HA antibodies revealed the expression of both constructs after the addition of Tetracycline, at their expected molecular weights. No leaky expression was observed in the uninduced parasite lines (Fig 2A). The western blots with the specific antibodies against *Tc*SIR2RP1 and *Tc*SIR2RP3 show a high degree of overexpression (20-fold) in the induced lines (Fig 2B). We also tested the inducible expression of the sirtuins in intracellular amastigotes and trypomastigotes by western blot of total lysates (Fig 3A) and immunofluorescense (Fig 3B) with anti-HA (quantification of results from Fig 3A are shown in S4 Fig). The tagged sirtuins are expressed only in the presence of Tetracycline, throughout *T*. *cruzi* life cycle.

### *Tc*SIR2RP1 is a cytosolic protein and *Tc*SIR2RP3 is mitochondrial

**Fig 4:**
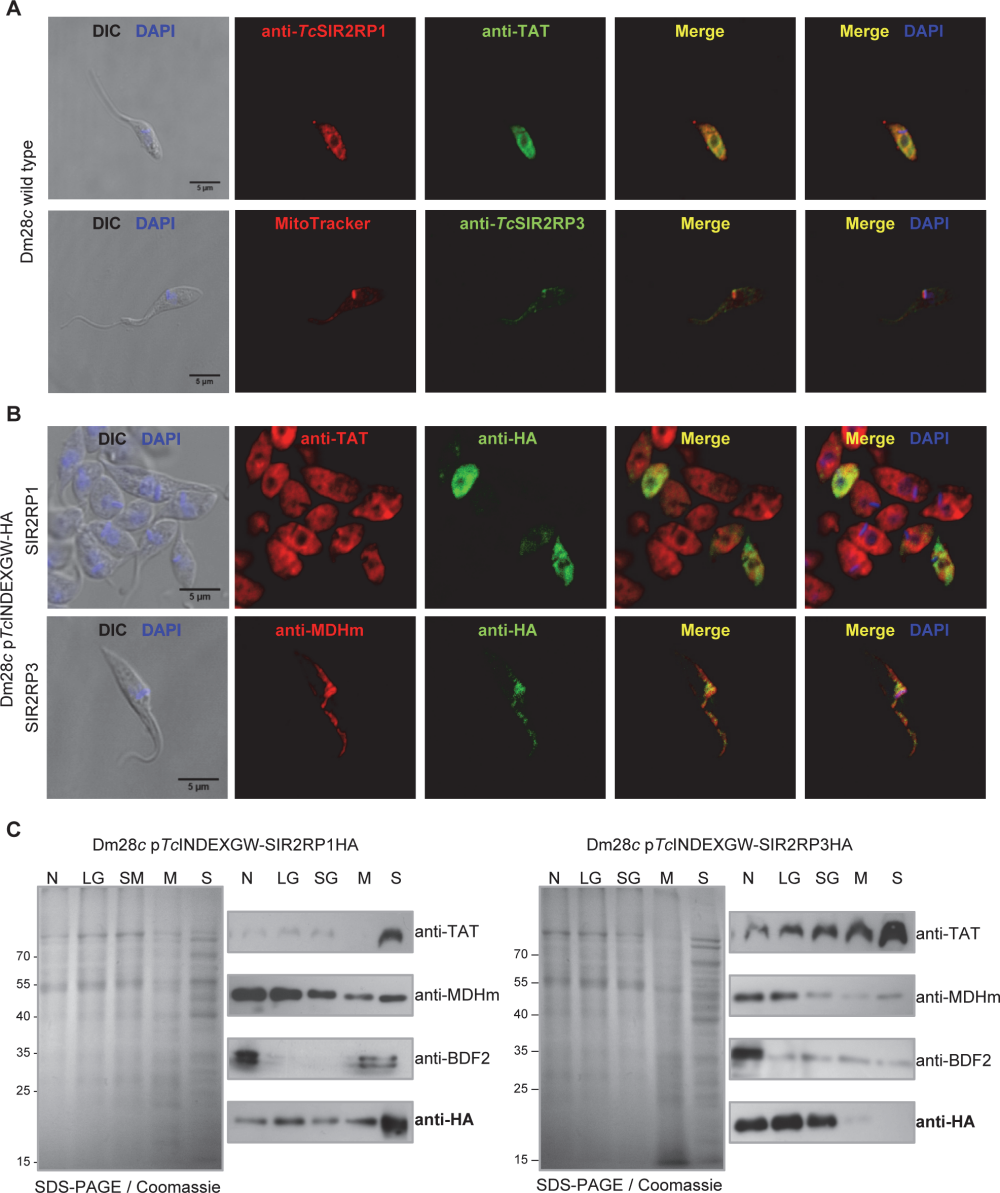
*Tc*SIR2RP1 localizes in the cytoplasm and *Tc*SIR2RP3 in the mitochondrion. (A) Immunofluorescence assay of Dm28*c* wild type epimastigotes using rabbit antibodies against *Tc*SIR2RP1 and *Tc*SIR2RP3 and cytosolic and mitochondrial markers (mouse anti-TAT and MitoTracker, respectively). Cy3-conjugated anti-rabbit (red) and FITC-conjugated anti-mouse and anti-rabbit (green) were used as secondary antibodies, and DNA was counterstained with DAPI (blue). Scale bar: 5 μm. (B) Immunofluorescence assay of Dm28*c* expressing *Tc*SIR2RP1HA and *Tc*SIR2RP3HA (0.25 μg/ml Tetracycline) using rat anti-HA and cytosolic and mitochondrial markers (rabbit anti-TAT and anti-MDHm). Cy3-conjugated anti-rabbit (red) and FITC-conjugated anti-rat (green) were used as secondary antibodies, and DNA was counterstained with DAPI (blue). Scale bar: 5 μm. (C) Western blot analysis of the subcellular fractionation of the induced lines obtained by differential centrifugation. Equal amounts of each fraction were loaded on SDS-PAGE (left panel) followed by western blot with anti-TAT (cytosolic marker), anti-MDHm (mitochondrial marker), anti-BDF2 (nuclear marker) and anti-HA. N, nucleus; LG, large granules; SG, small granules; M, microsomes; S, final supernatant.

Different technical approaches were performed to determine the localization of *Tc*SIR2RPs, using the specific antibodies raised in rabbit against the two recombinant proteins and commercial anti-HA monoclonal antibodies. Confocal immunocolocalization microscopies performed with cytosolic (anti-TAT) [34] and mitochondrial (MitoTracker) markers, together with anti-*Tc*SIR2RP1 and anti-*Tc*SIR2RP3, showed that *Tc*SIR2RP1 co-localizes with TAT (cytosol) and *Tc*SIR2RP3 with MitoTracker (Fig 4A). In parallel, the subcellular distribution of tagged sirtuins was analyzed by immunofluorescense of induced epimastigotes of each cell line with cytosolic (anti-TAT) and mitochondrial (anti-MDHm) markers together with anti-HA (Fig 4B). As can be observed in Fig 4B, SIR2RP1-HA colocalized with TAT and SIR2RP3-HA with MDHm, supporting the results obtained with specific antibodies.

To study in further detail their localizations, we performed subcellular fractionation by differential centrifugation of the transfected lines. The fractions obtained were analyzed by western blot with different subcellular markers (cytosolic TAT, mitochondrial MDH and nuclear BDF2 [35]), and with anti-HA (Fig 4C). The nuclear marker was enriched in fraction N, the mitochondrial marker in fractions N and LG, and the cytosolic marker in fraction S, as reported by Opperdoes et al [32]. In agreement with our previous results, SIR2RP1-HA exhibits a cytosolic pattern while SIR2RP3-HA exhibits a mitochondrial one.

### Deacetylation activity of *Tc*SIR2RPs

**Fig 5:**
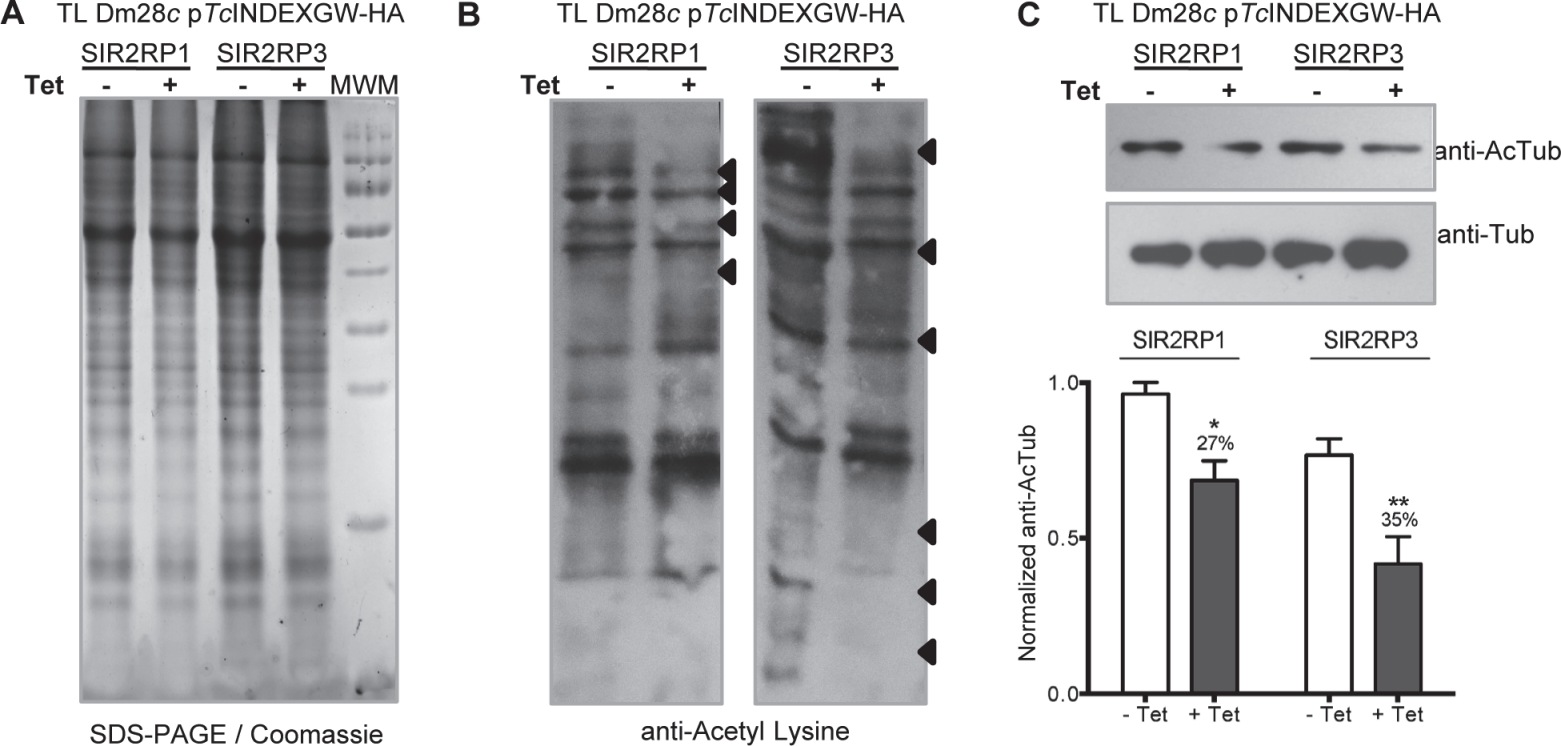
Overexpression of sirtuins changes the acetylation pattern in epimastigotes. Equal amounts of parasite lysate from each line in the absence (-, white bars) or presence (+, grey bars) of 0.25 μg/ml Tetracycline 24 hours post induction, were loaded on SDS-PAGE (A) followed by Western blot analysis using rabbit anti-Acetyl-lysine (B) and mouse anti-Tubulin and anti-Acetylated Tubulin antibodies (C). The deacetylated proteins in the anti-Acetyl-lysine western blot are depicted with black arrowsheads. The intensity of the acetylated-tubulin bands was quantified from n = 3 independent experiments and normalized to α-tubulin intensity. The bar graph represents the mean ± SEM; * p<0.05, ** p<0.005 (unpaired, two-tailed Student t test).

To test the deacetylation activity of *Tc*SIR2RPs, we performed western blot of uninduced and induced total lysates of each cell line with anti-Acetyl-lysine antibodies. Fig 5A shows the amount of protein loaded for each condition. Overexpression of both sirtuins reduced the acetylation levels of specific proteins (Fig 5B). The differentially acetylated proteins are depicted with arrowheads. It is worth noticing that the deacetylated proteins are different for each overexpressed sirtuin.

Alpha-tubulin is one of the most abundant acetylated proteins in trypanosomes. In fact, the rate of acetylated/non-acetylated α-tubulin in trypanosomatids is higher than in other eukaryotic cells like yeast or mammalian cells [36–37]. To test if any of the *Tc*SIR2RPs can deacetylate α-tubulin, we measured acetylated α–tubulin in uninduced and induced parasites by western blot analysis. When normalized to total α-tubulin, the results reflect significant diminutions of the acetylated form of the protein of 27% and 35% in *Tc*SIR2RP1HA and *Tc*SIR2RP3HA overexpressing lines respectively (Fig 5C). As already mentioned, deacetylation of α-tubulin mediated by SIR2RP1 was reported in *Leishmania*. However, even though our results are very confident, we consider that we cannot be conclusive enough to assign to *Tc*SIR2RP1 nor to *Tc*SIR2RP3 the function of being the *T*. *cruzi* tubulin deacetylase. The observed hypoacetylation could be an unspecific result due to the overexpression of a sirtuin. Furthermore, it has been demonstrated that in mammalian cells, tubulin is deacetylated not only by SIRT2, but also by the non-sirtuin deacetylase HDAC6 [38]. Since there are more than one deacetylases with similarity to HDAC6 in trypanosomatids, a deeper study of the whole set of deacetylase enzymes is needed to determine the most relevant tubulin deacetylase in this organism.

### *Tc*SIR2RP3 overexpression alters *T*. *cruzi* epimastigote growth

**Fig 6:**
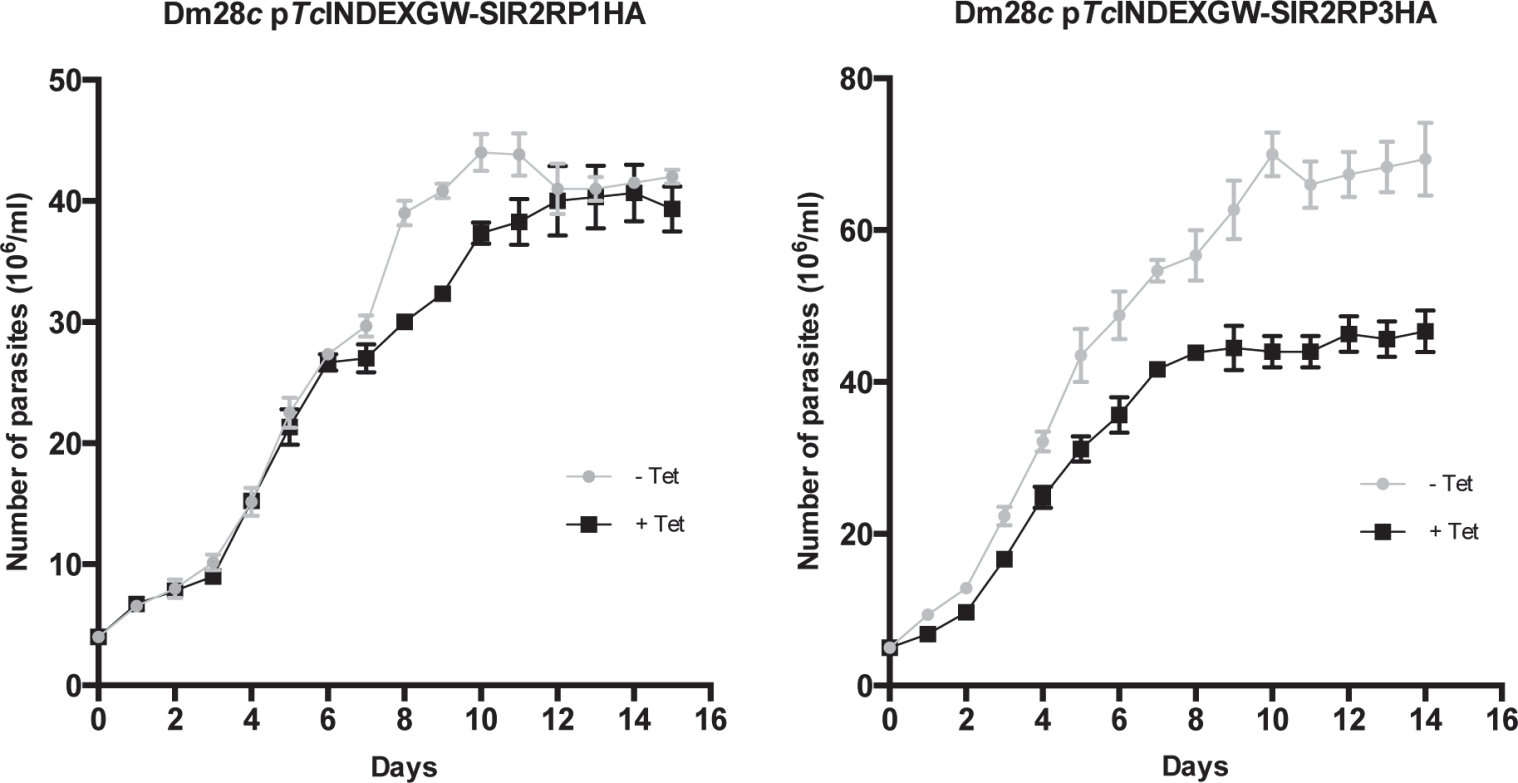
Effect of the overexpression of sirtuins on the growth rate of epimastigotes. Growth curves of epimastigotes transfected with p*Tc*INDEXGW-*Tc*SIR2RP1HA and *Tc*SIR2RP3HA in the absence (closed circles, grey line) or presence (closed squares, black line) of 0.5 μg/ml Tetracycline (which was re-added every 5 days) counted daily during 15 days. Results are representative of three independent experiments.

We monitored the effect of sirtuins overexpression on epimastigote growth by counting cell numbers daily after protein induction. Fig 6 shows that Dm28*c Tc*SIR2RP1HA cell line grew at similar rates in the absence and presence of Tetracycline (which was re-added every 5 days), but those harboring *Tc*SIR2RP3HA showed a delay in the growth rate when induced. Even more surprising is the fact that the *Tc*SIR2RP3HA expressing line reached stationary phase at a smaller number of parasites per ml. This culture continues in a stationary phase for the same period of time as the uninduced line. This phenomenon needs to be further studied in order to be completely understood, and the existence of a *quorum sensing* mechanisms recently described in *T*. *brucei* opens a novel possibility of interpretation [39].

### Sirtuins overexpression protects the parasite from the effect of sirtuin inhibitors

Sirtuins are considered as fundamental life sustaining biocatalysts and under various conditions were found to be pro-survival [40]. Therefore, there is an increasing interest in sirtuins as therapeutic targets. Several structures of complexes involving sirtuins and their inhibitors have been reported [17, 41]. In this work, we tested three sirtuin inhibitors: Nicotinamide (NAM) [42], Cambinol and Ex-527 [43]. All of them inhibited *T*. *cruzi* Dm28*c* epimastigotes growth in a concentration-dependent manner in axenic cultures. The IC_50_ values obtained for each inhibitor are shown in Table 1. Ex-527 IC_50_ (206.2 μM) is significantly higher compared to the other tested sirtuin inhibitors. Since, Ex-527 is a potent and selective *Hs*SIRT1 inhibitor, with a reported IC_50_ of 0.1–1 μM [43], the low toxicity we observed might indicate that neither of the sirtuins present in *T*. *cruzi* is related to *Hs*SIRT1, consistent with our phylogenetic analysis. On the contrary, NAM and Cambinol exhibited IC_50_ values similar to those obtained for purified sirtuins of other organisms such as: NAM IC_50_ for PfSIR2: 51.2 μM [44]; NAM IC_50_ for hSIRT5: 46.6 μM, hSIRT1: 50–100 μM, hSIRT3: 30 μM; Cambinol IC_50_ for hSIRT1: 56 μM, hSIRT2: 59 μM [45].

**Table 1:** IC_50_ * values of sirtuin inhibitors on *T*. *cruzi* Dm28*c* epimastigotes.

	Nicotinamide	Cambinol	EX-527
**Dm28*c* wild type**	61.33 μM	85.61 μM	206.2 μM
**Dm28*c* SIR2RP1 +Tet**	97.58 μM	135.8 μM	ND
**Dm28*c* SIR2RP3 +Tet**	81.72 μM	123.1 μM	ND

*IC_50_ was calculated after 72 hs culture.

ND: not determined.

**Fig 7:**
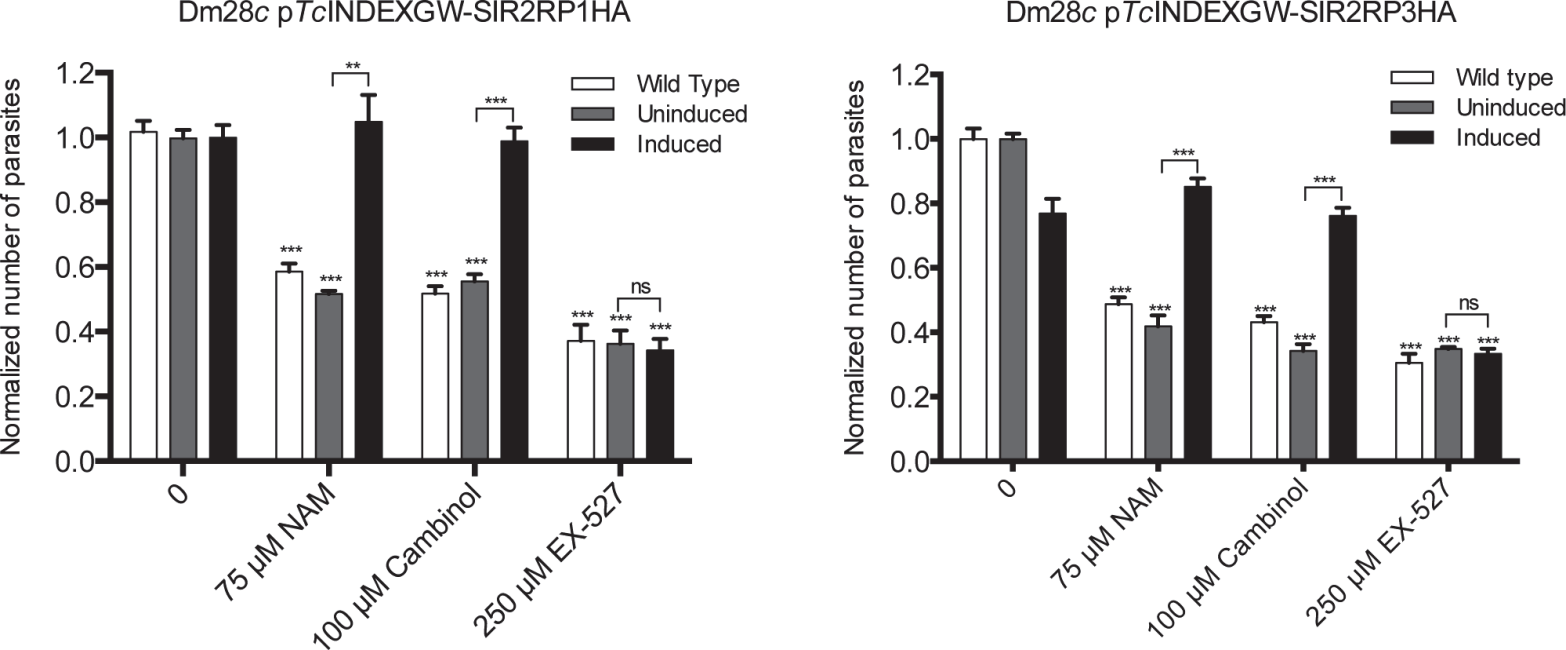
Overexpression of sirtuins protects epimastigotes from the growth defect caused by sirtuin inhibitors. Dm28*c* wild type (white bars) and uninduced (grey bars) and induced (black bars) epimastigotes of both transfected lines (Dm28*c* p*Tc*INDEXGW-*Tc*SIR2RP1HA and *Tc*SIR2RP3HA) were treated with three sirtuin inhibitors with concentrations above their IC_50_ values: 75 μM Nicotinamide, 100 μM Cambinol and 250 μM Ex-527. The experiment was performed in triplicates and cell growth was determined after culture for 72 hours by counting viable forms. The values obtained were normalized to the wild type growth without inhibitors. The growth rate of each transfected line with inhibitors was compared to the corresponding untreated one. We also compared the viability of the uninduced and induced overexpressing lines for each sirtuin inhibitor. The bar graph represents the mean ± SEM; * p<0.05, ** p<0.005, *** p<0.001 (unpaired, two-tailed Student t test).

We considered the possibility that sirtuin-overexpressing lines might be “protected” or less sensitive to the sirtuin inhibitors. To test this hypothesis, both parasite lines were treated with the inhibitors, in the absence and presence of Tetracycline. As seen in Fig 7, overexpression of sirtuins protects epimastigotes from the growth inhibition of NAM and Cambinol. Moreover, the treatment with these inhibitors reversed the growth defect of the induced *Tc*SIR2RP3HA cell line. In contrast, Ex-527-driven growth inhibition may be due to a pleiotropic effect and not to a reduction of the parasite’s sirtuin activities, since the inhibitory effect on trypanosomes is only observed at high concentrations. We also calculated the IC_50_ values of NAM and Cambinol for the overexpressing strains. The values obtained are shown in Table 1, as expected they are higher than for the wild type parasites.

### *Tc*SIR2RP1 overexpression stimulates the differentiation to metacyclic trypomastigotes

**Fig 8:**
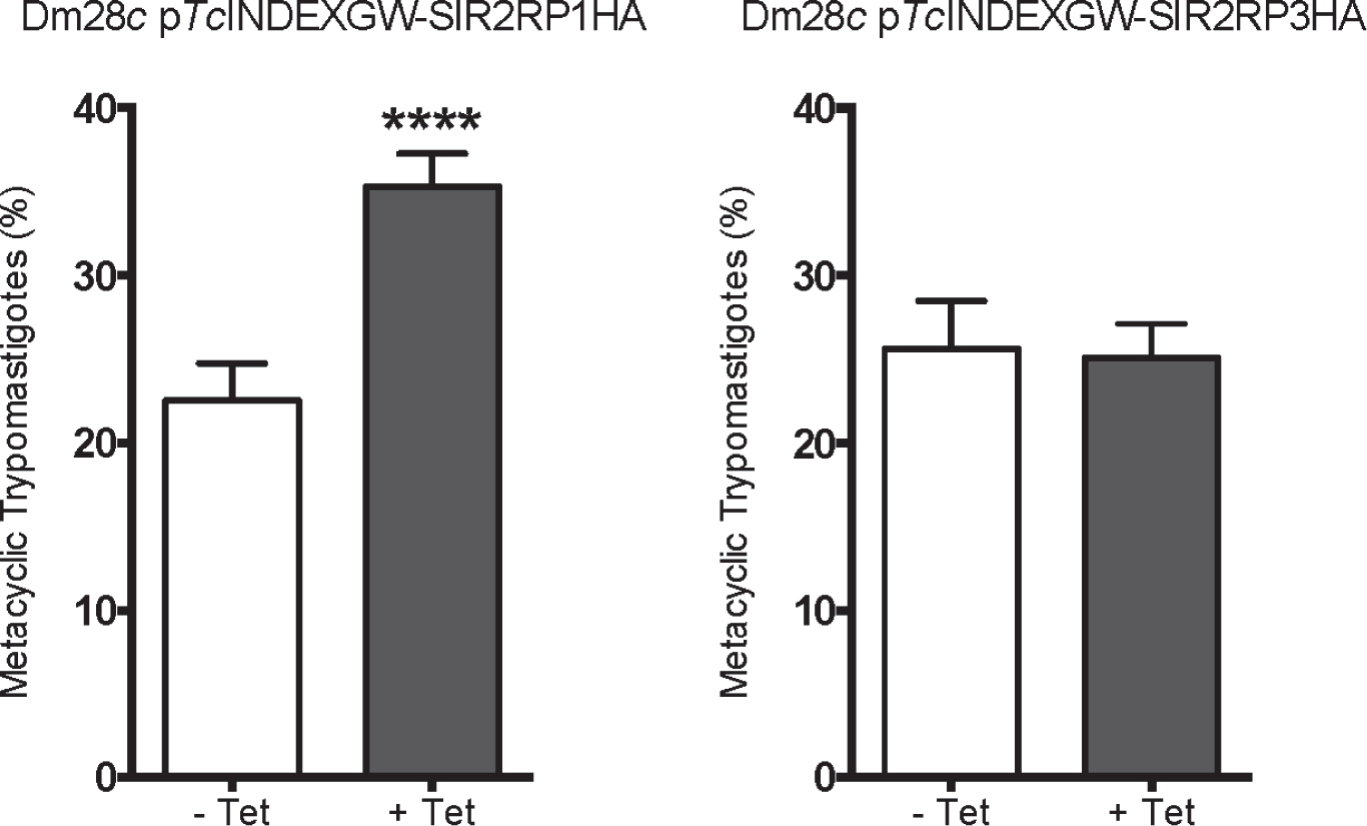
Overexpression of *Tc*SIR2RP1HA affects *in vitro* metacyclogenesis rate. *In vitro* metacyclogenesis using TAU medium of lines harboring transgenes encoding *Tc*SIR2RP1HA and *Tc*SIR2RP3HA uninduced (- Tet) or induced (+ Tet) with 0.5 μg/ml Tetracycline. The bar graph represents the mean ± SEM from three independent experiments; **** p<0.0001 (unpaired, two-tailed Student t test).

*In vitro* metacyclic trypomastigotes were produced from epimastigotes using TAU medium, in the absence (-Tet) or presence (+Tet) of Tetracycline. *Tc*SIR2RP1 overexpression resulted in a meaningful increase of metacyclogenesis (59%), whereas *Tc*SIR2RP3 strain showed similar levels of metacyclic trypomastigotes formation in both the induced and uninduced conditions (Fig 8).

### Effect of sirtuin overexpression on trypomastigotes infection of mammalian cells

**Fig 9:**
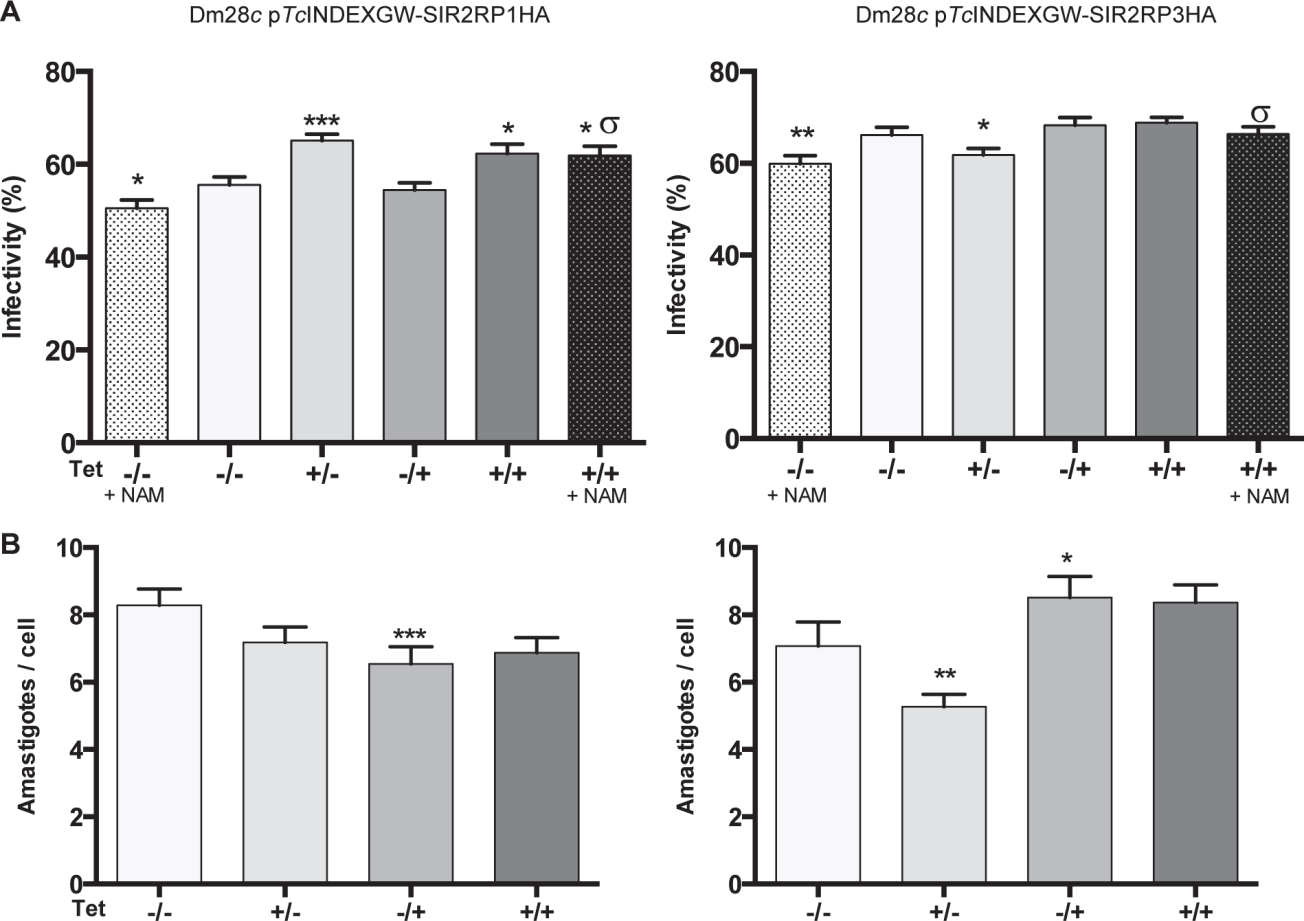
Sirtuin overexpression impacts on Vero cells infection. The infection and the post-infection incubation were performed in the absence or presence of 0.25 μg/ml Tetracycline: (-/-), Tet was never added to the medium; (+/-), trypomastigotes were pre-treated with Tet for 3 hours prior to infection, and it was added during the infection but not after; (-/+), trypomastigotes were not induced, Tet was only added for 48 hours post-infection at the amastigote stage; (+/+), trypomastigotes were pre-treated and Tet was present at all times. The conditions (-/-) and (+/+) were also tested in the presence of 75 μM Nicotinamide (+ NAM). The percentage of infected cells (A) and the number of amastigotes per cell (B) were determined by counting Giemsa-stained slides using a light microscope. Results are expressed as means ± SEM of triplicates, and represent one of three independent experiments performed. Each condition was analyzed by unpaired Mann-Whitney two-tailed Student t test with the control (-/-): * p<0.05, ** p<0.005, *** p<0.001. The same statistical analysis was used to compare uninduced and induced parasites in the presence of NAM (-/-, +NAM and +/+, +NAM): σ p<0.05.

To study the importance of sirtuins expression in trypomastigotes´ infectivity and in the replicative form present inside the mammalian host, we investigated how the transgenic lines induced with Tetracycline performed *in vitro* for invasion and replication in host cells. First, we performed the experiment with Dm28*c* wild type parasites to rule out any undesired effect of the Tetracycline treatment. Indeed, there was no significant difference in the infectivity rate nor in the number of amastigotes/cell (S5 Fig). Trypomastigotes were pre-incubated in the presence or absence of 0.25 μg/ml Tetracycline and NAM (100 μM) and then used to infect Vero cells at a ratio of 10 parasites per cell. After 6 h of infection at 37°C, the free trypomastigotes were washed out and replaced by complete medium alone or with Tetracycline (0.25 μg/ml) for 2 days post-infection. Microscopic observation of Vero cells stained with Giemsa showed that treatment of uninduced *T*. *cruzi* trypomastigotes with NAM [(-/-, +NAM) vs (-/-)] caused a significant reduction in the percentage of infected cells (as previously reported by Soares and coworkers [42]) (Fig 9A). The overexpression of both sirtuins protected the trypomastigotes from the negative effect of NAM [(+/+, +NAM) vs (-/-, +NAM)]. These results suggest that sirtuin activity is necessary for an effective infection of mammalian cells. To analyze the effect of sirtuin overexpression on the infectivity rate of trypomastigotes, we focused on the condition in which the expression was induced only in the trypomastigote stage during infection (+/-). As can be seen in Fig 9A, overexpression of *Tc*SIR2RP1HA increased the infectivity rate of trypomastigotes [(+/-) vs (-/-)], while overexpression of *Tc*SIR2RP3HA slightly diminished it [(+/-) vs (-/-)]. To test the effect of sirtuins overexpression on the proliferation of intracellular amatigotes (Fig 9B), we only added Tetracycline after the infection, when the trypomastigotes were washed out, for 48 hours post-infection (-/+). The number of amastigotes per infected cell is slightly decreased by the overexpression of *Tc*SIR2RP1HA, but increased by *Tc*SIR2RP3HA [(-/+) vs (-/-)]. The reduction in the number of amastigotes per cell observed when expression of *Tc*SIR2RP3 was induced during the infection [(+/-) vs (-/-)], might be a consequence of the diminished infectivity or it might indicate that the overexpression of this enzyme at the trypomastigote stage results in an inefficient differentiation to amastigotes, thus delaying the amastigotes´ replication. In contrast, when inducing at all times (+/+), the overexpression of amastigotes increases the proliferation rate hiding the trypomastigote to amastigote differentiation delay.

## Discussion

We present herein the first experimental characterization of *Trypanosoma cruzi* sirtuins *Tc*SIR2RP1 and *Tc*SIR2RP3. The expression of these enzymes is developmentally regulated throughout *T*. *cruzi* life cycle. *Tc*SIR2RP1 is highly expressed in epimastigotes and amastigotes, but at lower levels in trypomastigotes. On the other hand, *Tc*SIR2RP3 expression levels are higher in epimastigotes than in amastigotes, and it seems not to be expressed in trypomastigotes. The fact that the two sirtuins are differentially expressed along the life cycle of the parasite suggests that acetylation levels could play a role in the regulation of the biology of the parasite forms. It is remarkable that the life cycle pattern expression of Tetracycline induced/T7 transcribed sirtuins is similar to those of the wild type enzymes. This observation suggests that the protein levels could be regulated by a post-transcriptional mechanism independent of the 3´ and 5’ non-coding regions, which are absent in the p*Tc*-INDEX-GW constructions or by a post-translational mechanism.

Our results clearly demonstrate that *Tc*SIR2RP1 and *Tc*SIR2RP3 are, respectively, cytoplasmic and mitochondrial enzymes. These observations were expected, since both proteins lack the N-terminal portion responsible for the nuclear localization of *Sc*Sir2 and other related sirtuins. However, the possibility that under certain conditions, the trypanosomal sirtuins could be imported temporarily to the nucleus by an alternative targeting pathway cannot be completely ruled out. Taken together, our data suggest that sirtuin activity is important for the proliferation of *T*. *cruzi* replicative forms, for the host cell-parasite interplay, and for differentiation among life-cycle stages; but each one performs different roles in most of these processes.

Considering its cellular localization, *Tc*SIR2RP1 seems to be functionally more related to *Leishmania* than to *T*. *brucei* ortholog, even though *Tb*SIR2RP1 is more similar at the sequence level (68% identity) than *Lm*SIR2RP1 (55% identity). The fact that *T*. *brucei* has a nuclear sirtuin that is absent in the other TriTryps can be explained by some well-known differences existing among these species. *Tb*SIR2RP1 participates at the epigenetic-mediated silencing of RNA polymerase I-transcribed telomeric regions, but nothing similar occurs in *T*. *cruzi* or in *Leishmania*. In *Plasmodium falciparum*, a nuclear sirtuin (*Pf*SIR2A), phylogenetically unrelated to *Tb*SIR2RP1, is also implicated in telomeric gene silencing. Taken together, these results suggest that the participation of a sirtuin in histone deacetylation represents the exception, associated to telomeric gene silencing, rather than the rule of the function of sirtuins in these organisms, and it could be an example of convergent evolution.

In spite of their cellular localization and way of action, sirtuins are considered pro-survival regulators of metabolism and lifespan. These general functions are related with the use of NAD+ as substrate, which together with acetyl-CoA, the acetyltransferases substrate, are considered sensors of the energetic state of the cell. Nuclear sirtuins, like *Hs*SIRT1, regulate transcriptional response to starvation or redox stress and under certain conditions, cytoplasmic and mitochondrial sirtuins are imported to the nucleus with the same purpose (Reviewed in [46]). Since transcriptional regulation is absent in *T*. *cruzi*, it is reasonable to think that only non-nuclear functions will be found for these enzymes. One of the functions of cytoplasmic *Hs*SIRT2 is to deacetylate the enzyme phosphoenolpyruvate carboxykinase (PEPCK), increasing its stability, upon glucose deprivation [47]. Under caloric restriction, human mitochondrial sirtuins may also regulate gluconeogenesis from amino acids through glutamate dehydrogenase (GDH), an enzyme that converts glutamate to α-ketoglutarate, thereby controlling glucose production via the TCA cycle [48–50]. *Hs*SIRT3 and *Hs*SIRT4 modulate the activity of GDH through deacetylation and ADP-ribosylation, respectively (even though the existence of gluconeogenesis was only proved in amastigotes from Leishmania, it was already proposed that this pathway should be present in all trypanosmatids [51]). Human SIRT3 also decreases reactive oxygen species (ROS) production by stimulating superoxide dismutase 2 (SOD2), and enhances cellular respiration by increasing the activities of complex I, complex II (via succinate dehydrogenase (SDH)), complex III and isocitrate dehydrogenase 2 (IDH2), affecting both glucose and lipid metabolism. Finally, mitochondrial *Hs*SIRT3 stimulates β-oxidation and ketone body formation by targeting and activating long-chain acyl CoA dehydrogenase (LCAD) and 3-hydroxy-3-methylglutaryl-CoA synthase 2 (HMGCS2), respectively [52–53]. Although more specific research is needed to determine whether trypanosome sirtuins share these functions, some of our results can be interpreted under this general rationale: *Tc*SIR2RP1 improves metacyclogenesis, a differentiation process induced by starvation of the parasites [54–55]. The increased infectivity of this strain resembles that observed for *Lm*SIR2RP1 by Sereno and coworkers [56]. As this sirtuin was detected in the excreted/secreted material of parasites, they constructed *Lm*SIR2RP1-overexpressing fibroblasts, which were more permissive towards *Leishmania* invasion than control ones. These results suggest a function for parasite cytoplasmic sirtuins at the host-parasite interplay. However, the secretion of *Tc*SIR2RP1 is yet to be confirmed. In contrast, *Tc*SIR2RP3 seems not to be implicated in metacyclogenesis or cell infection but improves the replication of intracellular amastigotes, which occurs in an oxidant environment, suggesting the participation of this enzyme in redox stress response.

The activity of sirtuins in protecting cells from stress and starvation and so improving life span, led to the establishment of their role in cell proliferation. Currently, many sirtuin inhibitors are being assayed against different types of cancer. Conversely, sirtuin activators are considered as potential anti-aging drugs. It has also been proposed that sirtuins are promising targets for the development of anti-trypanosomal, anti-plasmodial and anti-leishmanial drugs and many well-characterized sirtuin inhibitors have already shown anti-parasitic activity [21–22, 40, 57]. In addition, an *in silico* structural and surface analysis of trypanosomal and human sirtuins determined potentially important structural differences in the corresponding inhibitor binding domains, indicating a possible selectivity of an inhibitor for a specific protein [58]. In a very recent *in silico* study, Sacconnay and coworkers [59] assayed a list of 50 phytochemicals previously described as anti-trypanosomals by docking into *Tc*SIR2RP1 and *Tc*SIRRP3, revealing that the activity of four of these compounds could be explained by the inhibition of the sirtuins activity. The results presented herein, contribute to the knowledge of these enzymes localization and function, and our *Tc*SIR2RP1HA and *Tc*SIR2RP3HA overexpressing *T*. *cruzi* strains can be useful tools for experimental screening of trypanosomatid sirtuin inhibitors.

## Supporting Information

S1 Fig Sirtuin sequence alignment. *Tc*SIR2RP1 and *Tc*SIR2RP3 were aligned with the seven human sirtuins (*Hs*SIRT1-7) and the Sir2 family founding member (*Sc*SIR2) using ClustalX2.1 and manually edited to highlight conserved and identical aminoacid residues based on BLOSUM 62 subsitution matrix data. The absence of the N-terminal sequence in both *T*. *cruzi* sequences and the characteristic C-terminal Ser-rich tract in *Tc*SIR2RP1 can be noted. The conservation in the core domain of Sir2 family, particularly the Cys residues from the Zn binding domain, and the GAG, NID and HG residues are indicated. Arrowheads indicate conserved critical catalitic and NAD^+^-binding residues according to crystal structures from other family members [60–61].
(TIFF)

S2 Fig Phylogenetic analysis. Unrooted phylogenetic tree of sirtuin sequences from Tritryp and diferent taxa (Reviewed in [24]). The tree was constructed using the Neighbor-Joining method with Clustal X2.1, with a random number generator seed of 150 and 5000 Bootstrap trials. Branch colours are used to show which taxa have sirtuin members for each of the previously described sirtuin groups. Only Tritryp sirtuins names are indicated.
(TIF)

S3 Fig Validation of the anti-*Tc*SIR2RP1 and anti-*Tc*SIR2RP3 specificity. Epimastigotes total lysates were fractioned in SDS-PAGE and transferred to nitrocellulose membranes. Transferred proteins were visualized with Ponceau S. Membranes were treated with 10% non-fat milk in PBS for 2 hours and then incubated with anti-*Tc*SIR2RP1 and anti-*Tc*SIR2RP3 rabbit polyclonal antibodies diluted 1/100 in 5% non-fat milk in PBS-Tween 0.01% for 16 hours at 4°C.
(TIF)

S4 Fig Quantification and normalization of the overexpression of *Tc*SIR2RP1HA and *Tc*SIR2RP3HA throughout *T*. *cruzi* life cycle. The anti-HA signal obtained for each developmental form in Fig 3A were quantified using ImageJ and normalized with the total protein load of its corresponding Coomassie stained lane.
(TIF)

S5 Fig Effect of Tetracycline treatment on the infection of Dm28*c* wild type parasites. The infection of Vero cells with wild type trypomastigotes was performed as described in Materials and Methods. The statistical analysis showed no significant difference.
(TIF)
