# Supplementary figures and images for: Overexpression of Cytoplasmic TcSIR2RP1 and Mitochondrial TcSIR2RP3 Impacts on Trypanosoma cruzi Growth and Cell Invasion
Source: PLoS Negl Trop Dis. 2015 Apr 15;9(4):e0003725. doi: 10.1371/journal.pntd.0003725 (PMC4398437; doi:10.1371/journal.pntd.0003725)

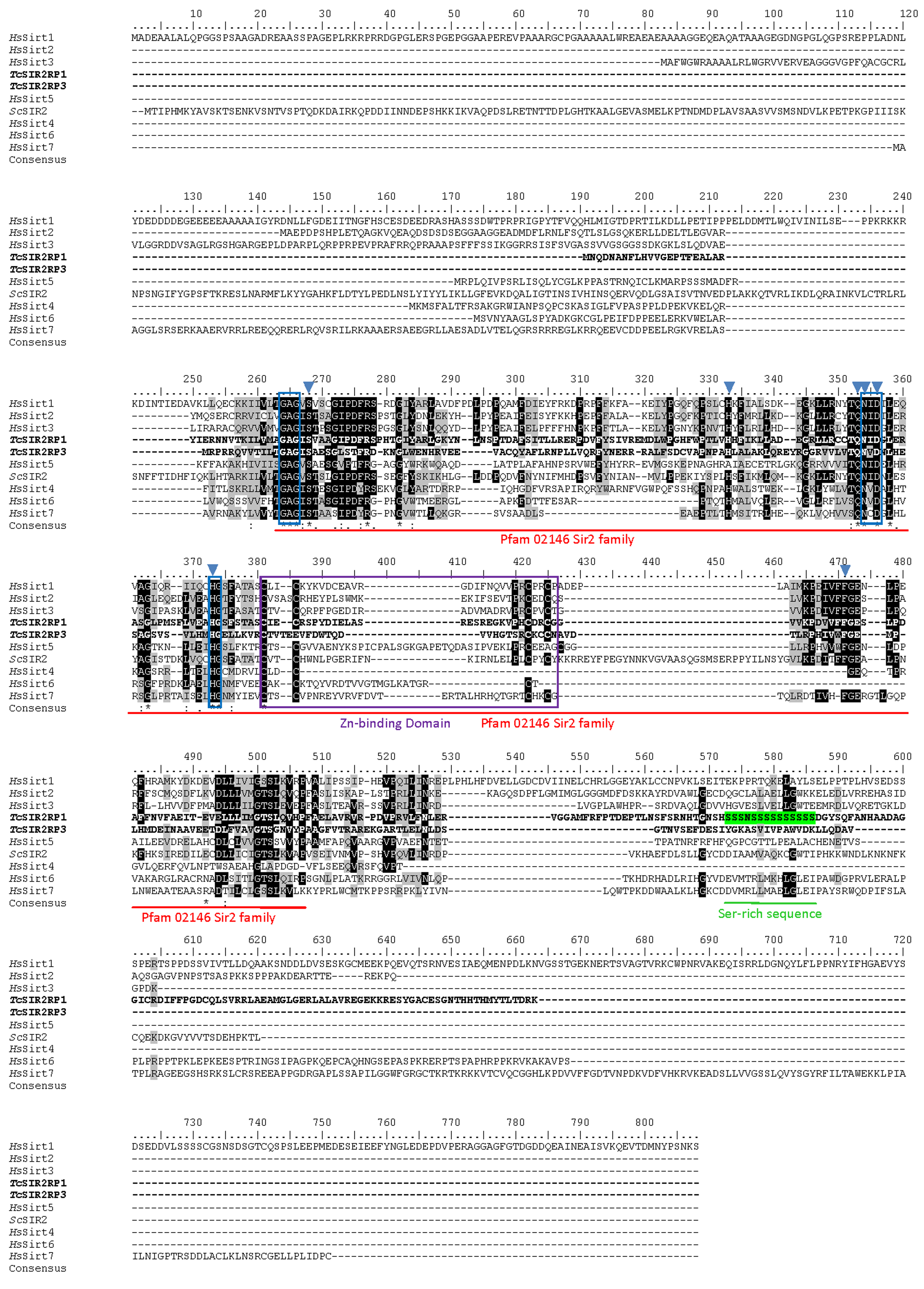

Supplement: S1 Fig — TcSIR2RP1 and TcSIR2RP3 were aligned with the seven human sirtuins (HsSIRT1-7) and the Sir2 family founding member (ScSIR2) using ClustalX2.1 and manually edited to highlight conserved and identical aminoacid residues based on BLOSUM 62 subsitution matrix data. The absence of the N-terminal sequence in both T. cruzi sequences and the characteristic C-terminal Ser-rich tract in TcSIR2RP1 can be noted. The conservation in the core domain of Sir2 family, particularly the Cys residues from the Zn binding domain, and the GAG, NID and HG residues are indicated. Arrowheads indicate conserved critical catalitic and NAD+-binding residues according to crystal structures from other family members [60–61]. (TIFF) [file pntd.0003725.s001.tiff]

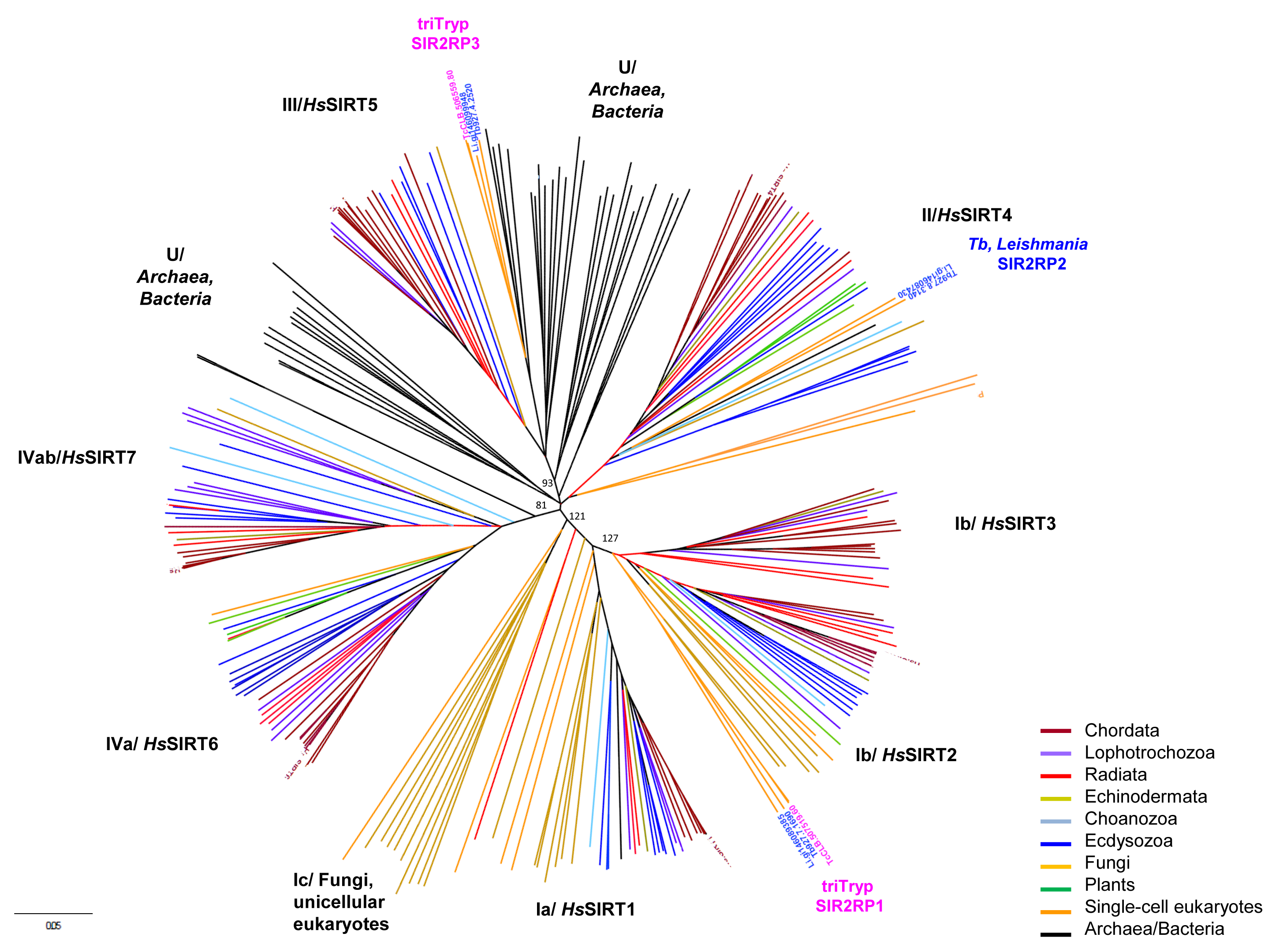

Supplement: S2 Fig — Unrooted phylogenetic tree of sirtuin sequences from Tritryp and diferent taxa (Reviewed in [24]). The tree was constructed using the Neighbor-Joining method with Clustal X2.1, with a random number generator seed of 150 and 5000 Bootstrap trials. Branch colours are used to show which taxa have sirtuin members for each of the previously described sirtuin groups. Only Tritryp sirtuins names are indicated. (TIF) [file pntd.0003725.s002.tif]

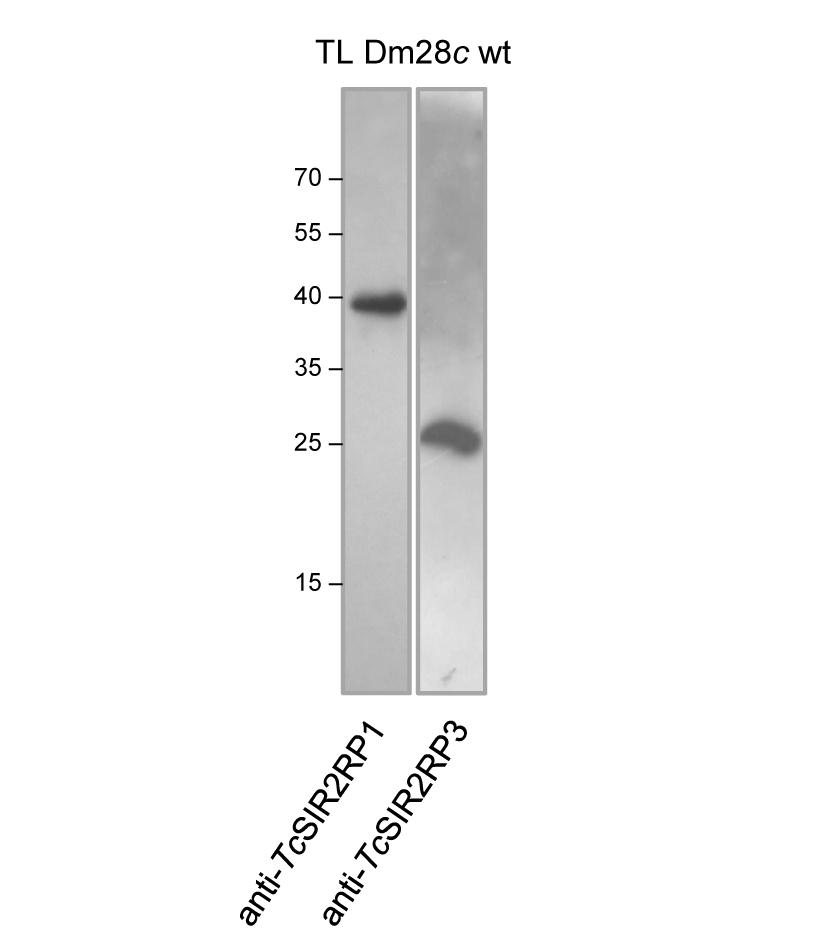

Supplement: S3 Fig — Epimastigotes total lysates were fractioned in SDS-PAGE and transferred to nitrocellulose membranes. Transferred proteins were visualized with Ponceau S. Membranes were treated with 10% non-fat milk in PBS for 2 hours and then incubated with anti-TcSIR2RP1 and anti-TcSIR2RP3 rabbit polyclonal antibodies diluted 1/100 in 5% non-fat milk in PBS-Tween 0.01% for 16 hours at 4°C. (TIF) [file pntd.0003725.s003.tif]

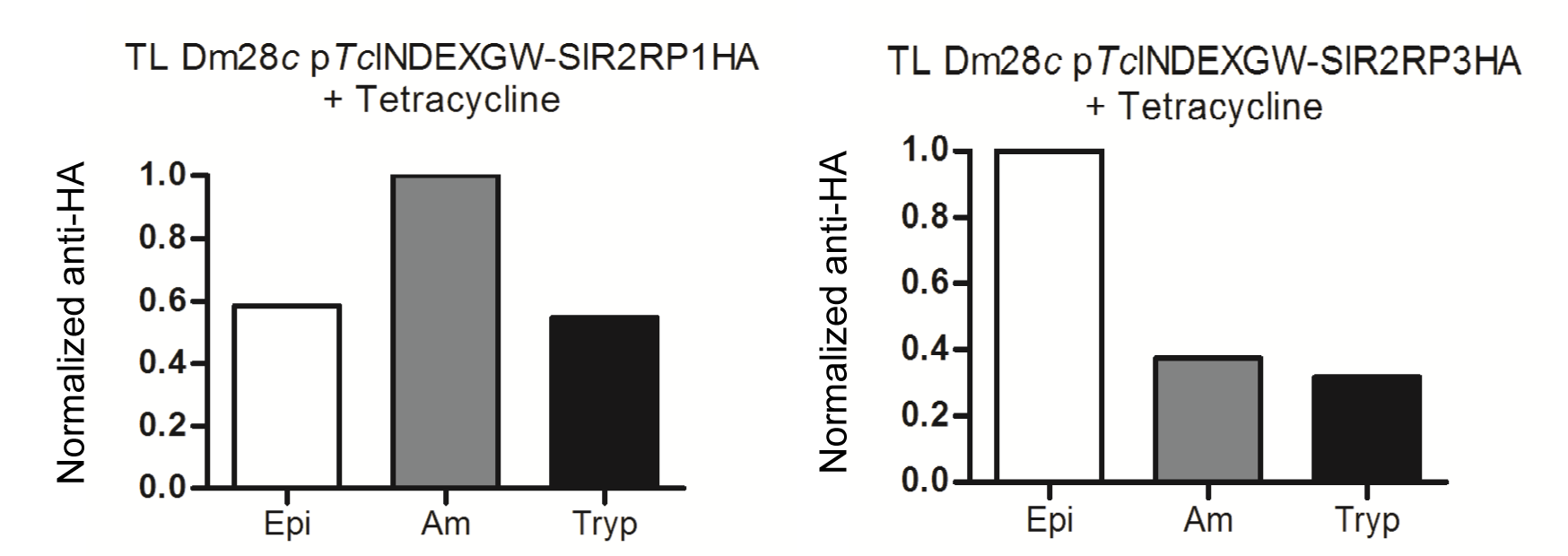

Supplement: S4 Fig — The anti-HA signal obtained for each developmental form in Fig 3A were quantified using ImageJ and normalized with the total protein load of its corresponding Coomassie stained lane. (TIF) [file pntd.0003725.s004.tif]

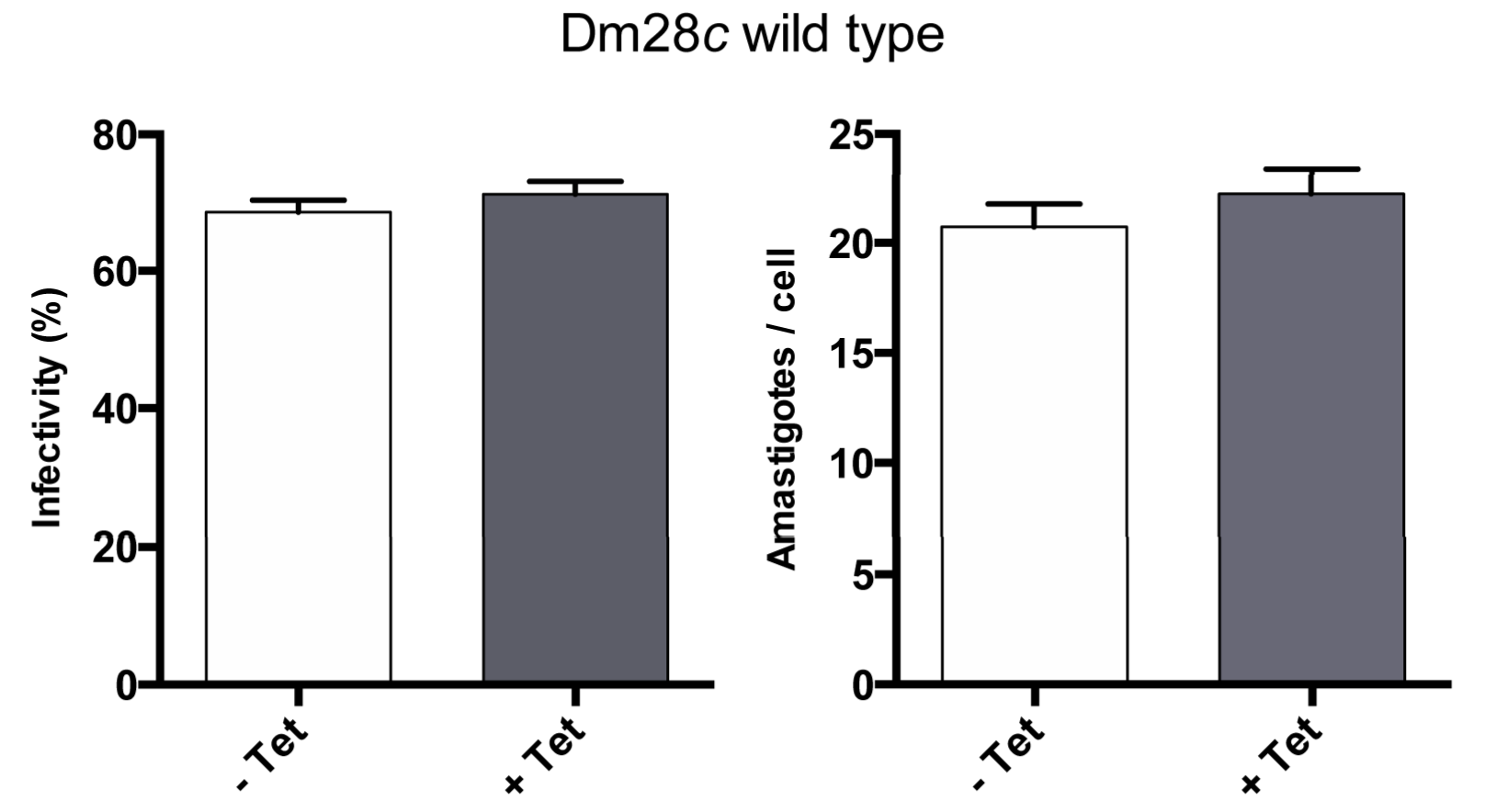

Supplement: S5 Fig — The infection of Vero cells with wild type trypomastigotes was performed as described in Materials and Methods. The statistical analysis showed no significant difference. (TIF) [file pntd.0003725.s005.tif]
